# Clinical Implications of (Pro)renin Receptor (PRR) Expression in Renal Tumours

**DOI:** 10.3390/diagnostics11020272

**Published:** 2021-02-10

**Authors:** Jon Danel Solano-Iturri, Enrique Echevarría, Miguel Unda, Ana Loizaga-Iriarte, Amparo Pérez-Fernández, Javier C. Angulo, José I. López, Gorka Larrinaga

**Affiliations:** 1 Department of Pathology, Donostia University Hospital, 20014 Donostia/San Sebastian, Spain; jondanel.solanoiturri@osakidetza.eus; 2 Department of Medical-Surgical Specialities, Faculty of Medicine and Nursing, University of the Basque Country (UPV/EHU), 48940 Leioa, Spain; 3 Biocruces-Bizkaia Health Research Institute, 48903 Barakaldo, Spain; joseignacio.lopez@osakidetza.eus; 4 Department of Physiology, Faculty of Medicine and Nursing, University of the Basque Country (UPV/EHU), 48940 Leioa, Spain; enrique.etxebarria@ehu.eus; 5 Department of Urology, Basurto University Hospital, University of the Basque Country (UPV/EHU), 48013 Bilbao, Spain; jesusmiguel.undaurzaiz@osakidetza.eus (M.U.); ana.loizagairiarte@osakidetza.eus (A.L.-I.); amparo.perezfernandez@osakidetza.eus (A.P.-F.); 6 Clinical Department. Faculty of Medical Sciences. European University of Madrid, 28905 Getafe, Spain; javier.angulo@universidadeuropea.es; 7 Department of Pathology, Cruces University Hospital, 48903 Barakaldo, Spain; 8 Department of Nursing, Faculty of Medicine and Nursing, University of the Basque Country (UPV/EHU), 48940 Leioa, Spain

**Keywords:** renal cell carcinoma, (Pro)renin receptor, renin–angiotensin system, prognosis

## Abstract

(1) Background: Renal cancer is one of the most frequent malignancies in Western countries, with an unpredictable clinical outcome, partly due to its high heterogeneity and the scarcity of reliable biomarkers of tumour progression. (Pro)renin receptor (PRR) is a novel receptor of the renin–angiotensin system (RAS) that has been associated with the development and progression of some solid tumours by RAS-dependent and -independent mechanisms. (2) Methods: In this study, we analysed the immunohistochemical expression of PRR at the centre and border in a series of 83 clear-cell renal cell (CCRCCs), 19 papillary (PRCC) and 7 chromophobe (ChRCC) renal cell carcinomas, and the benign tumour renal oncocytoma (RO, *n* = 11). (3) Results: PRR is expressed in all the tumour subtypes, with higher mean staining intensity in ChRCCs and ROs. A high expression of PRR at the tumour centre and at the infiltrative front of CCRCC tissues is significantly associated with high grade, tumour diameter, local invasion and stage, and with high mortality risk by UCLA integrated staging system (UISS) scale. (4) Conclusions: These findings indicate that PRR is associated with the development and progression of renal tumours. Its potential as a novel biomarker for RCC diagnosis/prognosis and as a promising therapeutic target should be taken into account in the future.

## 1. Introduction

Renal cell carcinoma (RCC) is one of the most common malignancies in Western Countries [1,2,3]. Nearly half of all cases of RCC are diagnosed in people more than 65 years old [2,4]. The incidence of RCC has been steadily increasing in recent decades, and it is expected that this trend will continue in the future due to the aging population of developed countries [4].

Clear-cell renal cell carcinoma (CCRCC) is by far the most frequent histological subtype of RCCs, accounting for approximately 75–80% of cases, followed by papillary renal cell carcinoma (PRCC) (10–15%) and chromophobe renal cell carcinoma (ChRCC) (5%) [5]. The most commonly accepted origin of CCRCC and PRCC is the proximal convoluted tubule of the nephron. ChRCC shares a common lineage with benign tumor renal oncocytoma (RO) (5%) and both seem to originate from the intercalated cells of the distal nephron [6]. CCRCC is the most aggressive of the RCCs; 30% of patients are metastatic at diagnosis and another 30% of patients with localized disease eventually progress to metastatic disease [5]. A close correlation between some specific genomic signatures and clinical aggressiveness has been detected in recent studies [7], but an easier identification of changes linked to tumour behaviour and clinical outcome is necessary to reach an efficient improvement in the management of CCRCC patients [8]. With this regard, in recent years, novel prognostic biomarkers and therapeutic approaches have been explored. Thus, study of the components of the renin–angiotensin system (RAS) in the context of renal neoplasms and the potential usefulness of drugs that target this peptidergic system has become a promising research field for RCC [9].

The RAS has been traditionally described as a circulating hormone system that regulates cardiovascular and renal function [10]. However, RAS is also locally expressed in several organs and tissues and its paracrine, autocrine, and intracrine signals can regulate long-term biologic processes such as cell growth [9,11]. Thus, the discovery of intrarenal RAS has been crucial to understanding its involvement in non-neoplastic chronic kidney disease [9,12]. For years, angiotensin-converting enzyme (ACE) and angiotensin-II receptor (AT1R) inhibitors (ACEis and ARBs) have been widely used in the management of this pathology. The mechanism of action of these drugs is based on the inhibition of the ACE/Ang-II/AT1R axis, which induce cell proliferation, fibrosis and inflammation [9,12].

These phenomena are also part of neoplastic processes and, therefore, the study of RAS and the potential of RAS-targeting therapies has received considerable attention in research into renal cancer [9,11]. Thus, imbalances in components of the intrarenal RAS, such as the up-regulation of AT1Rs in RCC cells and ACE in tumour vessels, have been associated with renal cancer development and progression [13,14,15]. Besides, the use of the abovementioned RAS inhibitors (RASis) has been associated with better response to current treatments and better outcomes of patients with metastatic RCC [16,17,18].

The (pro)renin receptor (PRR) is a novel component of the RAS that was first described in the past decade [19] and that is expressed in several organs and tissues, including the kidney [12]. The most well-known function of this protein is the activation of RAS. PRR binds renin enzyme and its inactive precursor prorenin, which enhances their activity and the production of angiotensin I, which is converted by ACE in angiotensin II, leading to AT1R-mediated signals. PRR is also activated after the binding of (pro)renin, which leads to PI3/AKT/mTOR and MAPK/ERK signalling [12,20,21]. Besides this, PRR is considered to function as a hinge molecule between the Wnt receptor and the V-ATPase that mediates Wnt receptor internalization and the subsequent Wnt/β–catenin signaling [12,21].

These RAS-dependent and -independent signalling pathways contribute to cancer initiation, so it was expected that PRR expression could be altered in tumour tissues [21]. Thus, increases in this protein have been described in pancreatic ductal adenocarcinoma [22,23], glioma [24,25], colorectal [26,27], breast [28] and endometrial cancer [29]. Taking into account that PRR exerts important functions in kidney physiology and that it takes part in inflammatory and fibrotic processes of this organ [12], changes in this protein can also be expected in kidney neoplasms. The Cancer Genome Atlas (TCGA) described high mRNA levels of PRR in RCCs when compared with the uninvolved part of the kidney [21]. The Human Protein Atlas (https://www.proteinatlas.org (accessed on 5 February 2021)) described PRR staining in RCCs; however, the analyses were limited to only 11 cases, which was insufficient to understand the association between this protein and tumour progression.

In this study, we analysed PRR immunohistochemical expression in a series of 120 kidney tumours. The series included three subtypes of RCC (CCRCC, PRCC and ChRCC) and the benign tumour RO. Both the centre of the tumour and the infiltration front was analysed to test the possible heterogeneity of PRR expression in these tumours. Since CCRCC is the most frequent RCC [5], we analysed the association between PRR and tumour progression and its impact on the prognosis of CCRCC patients.

## 2. Materials and Methods

The present study, including all its experiments, comply with current Spanish and European Union legal regulations. The Basque Biobank for Research (OEHUN) (www.biobancovasco.org (accessed on 5 February 2021)) was the source of samples, and the data from employed patients could possibly be used for research purposes. Each patient signed a specific document which was approved by the Ethical and Scientific Committees of the Basque Country Public Health System (Osakidetza) (PI + CES-BIOEF 2018-04).

### 2.1. Patients

A total of 120 renal tumours, surgically removed at Basurto University Hospital between 2012 and 2016, were collected for the study: 83 CCRCCs (mean age: 61.9 years, 58 males and 25 females), 19 PRCCs (mean age: 53.5 years, 15 males and 4 females), 7 ChRCCs (63.9 years, 6 males and 1 female) and 11 ROs (mean age: 63.4 years, 4 males and 7 females). Samples from the tumour centre and the infiltrating front were included in tissue microarrays (TMAs) for further immunohistochemical analyses. One sample of the uninvolved kidney was also included in each TMA.

Table 1 summarizes the pathological and clinical characteristics of patients with CCRCC. American Joint Committee on Cancer (AJCC) [30] and WHO/ISUP [31] methods were applied to assign tumour, node and metastasis (TNM) Stage and Grade, respectively.

### 2.2. Immunohistochemistry

Rabbit polyclonal antibody specific for PRR [ref. HPA003156; Sigma-Aldrich (Saint Louis, MO, USA) at 1/50 dilution] was used for the immunostaining of formalin-fixed and paraffin-embedded tumour tissues. The antibody’s specificity was tested previously [27] and the immunostaining process was performed following routine methods in an automatic immunostainer (Dako Autostainer Plus, Dako-Agilent, Santa Clara, California, USA). Briefly, antigen retrieval was carried out in a low-pH buffer (K8005, Dako, Santa Clara, California, USA) for 20 min at 95 °C. The samples were incubated with the primary antibody for 50 min at room temperature. Then, the primary antibody was washed and samples were incubated for 20 min with secondary anti-rabbit antibody (K8021, Dako). EnVision-Flex detection system together with an HRP-enzyme-labelled polymer (SM802, Dako) was employed. A positive reaction was visualized with diaminobenzydine (DAB) solution (DM827, Dako), followed by counterstaining with haematoxylin (K8008, Dako).

Slides were reviewed under light microscopy for staining evaluation. Two observers independently evaluated the slides and, in the event of discrepancies, samples were re-evaluated to arrive at a final conclusion. As previously reported [27], staining patterns were scored as negative, weak and intense cytoplasmic and membranous staining.

### 2.3. Statistical Analysis

The statistical analysis was performed with SPSS^®^ 24.0.

We applied a Kolmogorov–Smirnov test to assess if data obtained from tumour tissues followed a normal distribution. Based on this information, data were further analysed using parametric or non-parametric tests.

The correlation between PRR expression in tumour centre and front and patient age and gender was evaluated using Spearman Rho tests. Associations between categorical PRR expression (weak/intense) in renal tumour tissues’ pathological and clinical variables were tested by Chi-square (χ2) test. Cancer-specific survival (CSS) analysis was performed by long-rank test with a cut-off point based on the categorical expression of PRR.

## 3. Results

### 3.1. Kidney Tumours Express PRR

**Figure 1:**
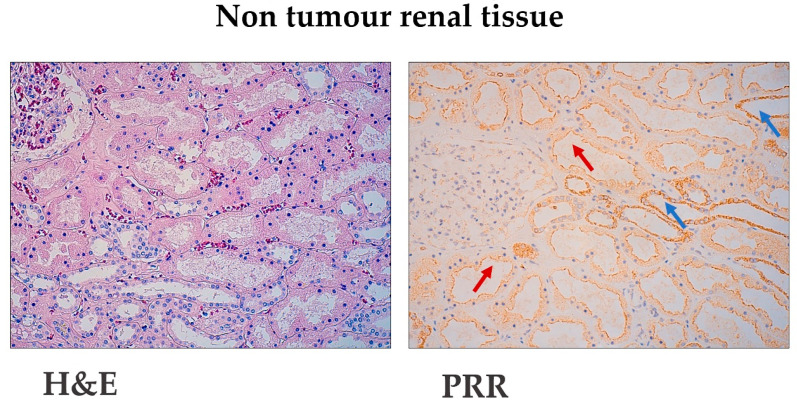
Immunohistochemical (Pro)renin Receptor (PRR) staining in non-tumour renal tissue. Hematoxylin–Eosin and PRR immunostaining at the uninvolved part of the kidney. Original magnification, ×250. Red arrows point to proximal tubules with weak PRR expression. Blue arrows point to distal nephron tubules with intense PRR staining.

The uninvolved part of the kidney showed more intense staining in tubules from the distal nephron than from proximal ones (Figure 1). PRR staining is distributed as cytoplasmic granular staining of renal cells and concentrates close to the cell membrane. This distribution corresponds both to the cell-surface and the cytoplasmic organelles.

**Figure 2:**
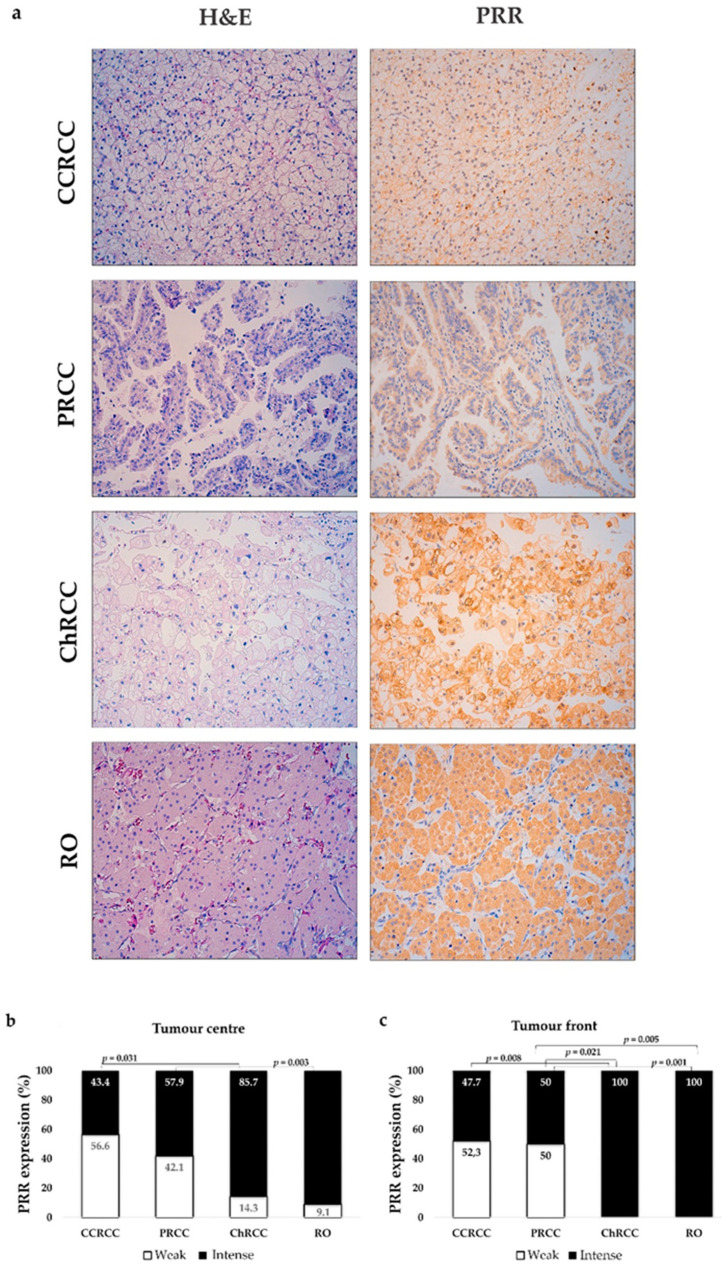
Immunohistochemical PRR staining in kidney tumours. (**a**) PRR immunostaining of different tumor subtypes (clear cell renal cell carcinoma (CCRCC); papillary renal cell carcinoma (PRCC); chromophobe renal cell carcinoma (ChRCC); renal oncocytoma (RO)). Original magnification, ×250. PRR staining intensity both at centre (**b**) and front (**c**) of tumours was grouped as weak and intense. ChRCCs and RO showed intense PRR staining, whereas CCRCC and PRCC showed weak staining. Chi-square test was used for data analysis. H&E: Hematoxilin–Eosin.

Same as has been observed in normal renal tubules, the different subtypes of renal cell carcinomas (CCRCC, PRCC and ChRCC) and RO expressed PRR in the cytoplasm and plasmatic membrane of tumour cells (Figure 2a). Only one case in all the series was PRR negative, so data from IHC analysis are represented by two values: weak and intense.

Nearly half of CCRCCs and PRCCs showed weak staining of PRR, whereas in ChRCC and RO, the staining was mainly intense. Significant differences were found between tumours from distal and proximal nephron both in the centre and tumour front (Figure 2b,c).

We also compared differences in the expression of PRR between the tumour centre and front in each kidney tumour subtype. Both tumour locations showed a similar staining pattern in CCRCC (Chi-square test, *p* = 0.6), PRCC (*p*= 0.64), ChRCC (*p* = 0.3) and RO (*p* = 0.31).

### 3.2. PRR Expression in CCRCC Changes Depending on Tumour Aggressiveness

Data from CCRCC tumours were stratified by pathological parameters that were tightly related to tumour aggressiveness such as WHO/ISUP histological grade, tumour necrosis, tumour size, local invasion (pT), the presence/absence of affected lymph nodes (N) and metastases (M), TNM stage and tumour progression (SSIGN scale). The association between PRR and clinical variables such as patients’ sex, age, mortality risk (UISS scale), and 5-year cancer-specific survival was also analysed.

The non-parametric Rho Spearman test was performed to assess if PRR protein expression varies according to the gender or age of the patients. Results showed no statistically significant correlation between PRR and sex (expression in tumour centre: Spearman Rho, r = 0.061, *p* = 0.58; tumour front: r = −0.01, *p* = 0.99) and age (expression in tumour centre: r= 0.176, *p* = 0.11; tumour front: r= 0.183, *p* = 0.12).

#### 3.2.1. PRR Expression is Higher in High Grade CCRCCs

**Figure 3:**
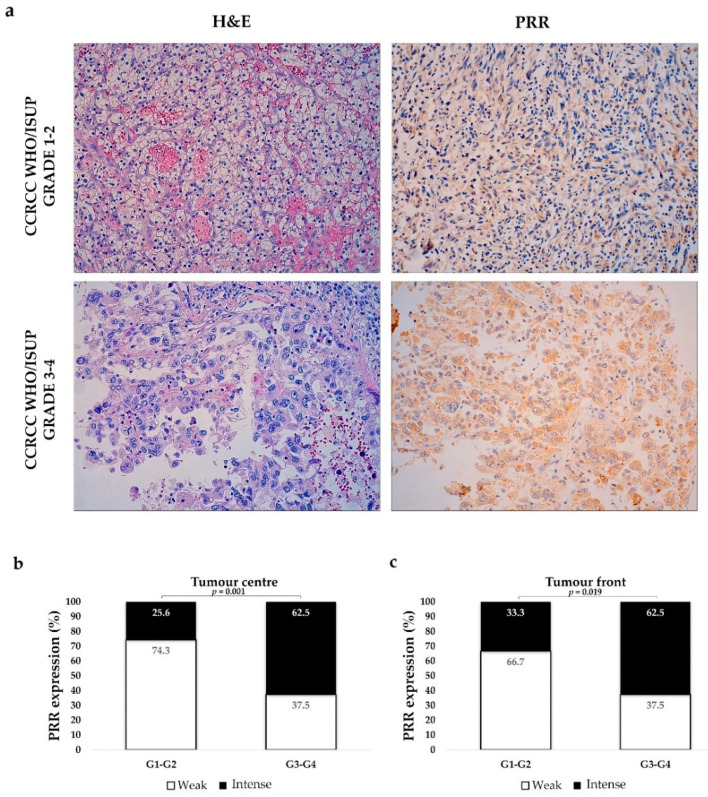
PRR expression according to tumour grade. Immunostaining at the tumour centre of low- and high-grade CCRCCs (**a**). Original magnification, ×250. H&E: Hematoxilin–Eosin. PRR staining intensity both at centre (**b**) and front (**c**) of tumours was grouped as weak and intense. Staining was more intense in high-grade CCRCCs. Chi-square test was used for data analysis.

We stratified cases as low- (G1–G2) and high histological grade (G3–G4). High-grade CCRCCs expressed significantly stronger PRR staining than low-grade tumours, both at the centre and at the infiltrating front (Figure 3).

#### 3.2.2. PRR Staining is Stronger in Large Tumours

**Figure 4:**
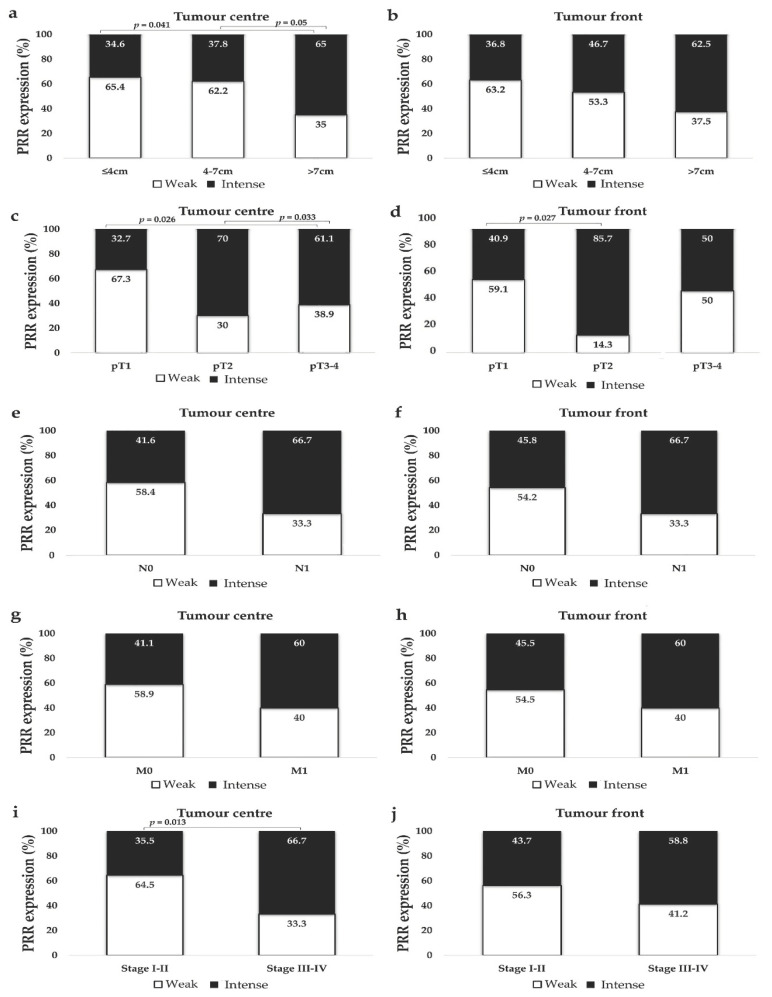
Immunohistochemical PRR staining according to CCRCC tumour size (**a**,**b**), local invasion (pT) (**c**,**d**), lymph node (N) (**e**,**f**) and distant metastasis (M) (**g**,**h**), and TNM Stage (**i**,**j**). PRR staining intensity was grouped as weak and intense. Chi-square test was used for data analysis. N0: No lymph node metastasis; N1: lymph node metastasis; M0: No distant metastasis; M1: distant metastasis.

CCRCCs were classified in three groups following previously reported classifications [27,32]: tumours with 4 cm or smaller, 4 to 7 cm, and larger than 7 cm. PRR staining was more intense in larger CCRCCs than in small or medium ones. The difference was statistically significant at the centre of the tumour (Figure 4a,b).

#### 3.2.3. PRR Expression is Higher in CCRCCs with Higher Local Invasion (pT)

The series had a limited number of pT4 cases and we stratified local invasion in three groups: pT1 (organ-confined tumours ≤7 cm), pT2 (organ-confined tumours >7 cm) and pT3-pT4 (non-organ-confined tumours). PRR expression at the centre and at the infiltration front of pT2 tumours was significantly higher than in pT1 ones. Non-organ-confined tumours also showed stronger staining of PRR than pT1 ones, with significant results at the tumour centre (Figure 4c,d).

#### 3.2.4. PRR Expression Does Not Vary Significantly in Metastasized and Not Metastasized CCRCCs

Tumours that invaded locorregional lymph nodes (N1) showed a similar trend, towards more intense PRR staining than tumours without lymph node invasion (N0), but data were not statistically significant (at tumour centre, Chi-square *p* = 0.23; at tumour front, *p* = 0.33) (Figure 4e,f). Similarly, PRR expression was higher in CCRCCs with distant metastasis (M1) than in tumours without, but not significantly (at tumour centre, Chi-square *p* = 0.23; at tumour front, *p* = 0.39) (Figure 4g,h).

#### 3.2.5. PRR Expression is Significantly Higher in High Stage Tumours

CCRCCs were stratified as low-stage (Stage I–II) and advanced- or high-stage (Stages III–IV). PRR staining at the centre of tumours was stronger in advanced than in low-stage CCRCCs (Figure 4i,j).

#### 3.2.6. PRR Expression is Higher in CCRCC Patients with Intermediate and High Mortality Risk (UISS)

We used two validated scales that predict tumour progression and mortality risk of CCRCC patients: the Mayo Clinic stage, size, grade, necrosis (SSIGN) model [33] and the UCLA Integrated Staging System (UISS) model [34]. SSIGN scale was stratified in two groups (low vs. high progression), and UISS was classified in three groups (low vs. intermediate vs. high mortality risk). We also had data of patients’ 5-year cancer-specific survival (CSS). The association between SSIGN (Log-rank test, *p* = 0.026) and UISS scales (Log-rank test, *p* = 0.013 × 10^−5^) and 5-year CSS was statistically significant.

**Figure 5:**
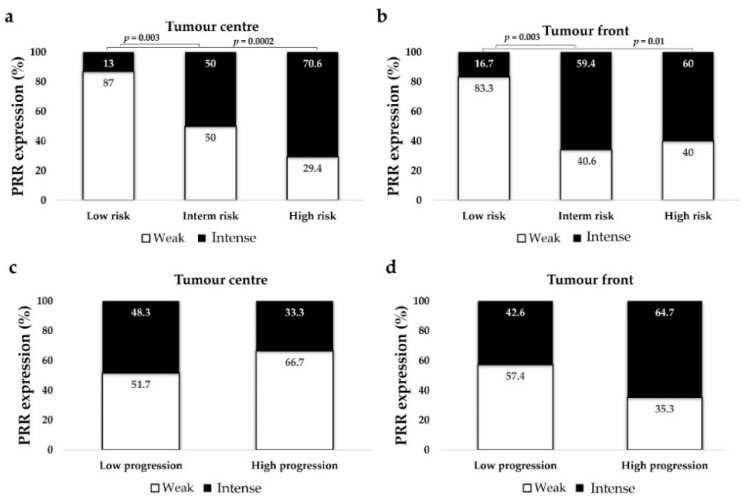
Immunohistochemical PRR staining according to UISS (mortality risk) (**a**,**b**) and SSIGN (disease progression) scales (**c**,**d**). PRR staining intensity was grouped as weak and intense. Chi-square test was used for data analysis.

PRR at the tumour centre and front showed more intense PRR staining in patients with intermediate and high mortality risk (UISS) than in those with low risk (Figure 5a,b). However, this expression was not associated to the SSIGN scale (PRR at tumour centre, Chi-square test *p* = 0.23; PRR at tumour front, *p* = 0.12) (Figure 5c,d), or to the 5-year CSS of these patients (PRR at tumour centre, Log-rank test *p* = 0.381; PRR at tumour front, *p* = 0.857).

## 4. Discussion

RCC is a complex and heterogeneous tumour with only two first-line therapeutic approaches with some efficacy at present: the inhibition of angiogenic pathways by tyrosine-kinase inhibitors (TKIs) and the improvement in anti-tumour immunity by recently included immune checkpoint inhibitors (ICIs) [35]. Clinical data demonstrated that the concomitant use of RASis improves the response to TKI treatment in metastasic RCC patients [16,17,18]. Preclinical studies supported these data, demonstrating a “hypertrophy” of the ACE/AngII/AT1R axis in RCC tissues and proangiogenic and proinvasive effects of AT1R stimulation by AngII [9,13,14,15]. Moreover, it has been demonstrated that AngII/AT1R-signaling contributes to an immunosuppressive microenvironment in different ways and that RASis are promising drugs to improve the response to ICIs [36].

The second most-known axis of the RAS, the ACE2/Ang1-7/Mas axis, counterbalances the actions of AngII. Anti-tumour effects of this axis have been demonstrated in several solid cancers [9,11]; however, its role in RCC development is more controversial [9,11]. On the one hand, it was shown that Ang1-7 induced protumour and proinvasive phenomena in RCC cell lines through Mas/AKT-signalling pathways [37] and that ACE2 protein expression was higher in high-grade CCRCCs [15]. On the other hand, it has recently been described that higher *ACE2* mRNA levels in CCRCC are associated with better survival of RCC patients and better response to immunotherapy [38].

The place of PRR in the complex scheme of RAS and renal cancer remains to be determined. As previously reported [12], we observed that this protein is expressed in proximal tubules and in the distal nephron of the uninvolved kidney. Both are topographic origins of the main renal tumours [5] and, as expected, PRR is expressed in three subtypes of RCCs. Besides this, the staining of this protein was more intense in CCRCCs with high histological grade, diameter and stage, and was associated with a higher risk of mortality according to the UISS scale. These results agree with studies carried out in other solid tumours, showing the potential of PRR as a biomarker of worse prognosis [21]. Furthermore, we reported recently that PRR staining was not very different in the centre and front of colorectal cancers [27], a result that was repeated in RCCs. This finding suggests that the intrinsic heterogeneity of these tumours may not affect their analysis, which is favourable for the potential use of PRR as an immunohistochemical prognostic biomarker [27].

The mechanism by which PRR upregulation could affect the progression of kidney could be RAS-dependent or -independent [12,21]. It is known that under pathologic conditions, PRR induces AngII/AT1R-dependent secretion of pro-fibrotic and pro-inflammatory cytokines in cells from the proximal and distal nephron [12]. Treatment with the renin inhibitor aliskiren demonstrated antiproliferative effects of RCC [39]; therefore, it could be hypothesized that the upregulation of PRR could enhance renin activity, contributing to the stimulation of ACE/AngII/AT1R axis in RCC tissues.

The binding of renin or prorenin to PRR can activate the PI3/AKT/mTOR pathway independently of the action of angiotensins [12,21]. This is a well-known oncogenic pathway, which is aberrantly active in CCRCC [40]. Furthermore, PRR is also an important component of the Wnt receptor complex and acts as an adaptor between LRP6 and the V-ATPase. This facilitates the binding of Wnt ligands to the Wnt receptor complex and the activation of the Wnt/β-catenin signalling [12,21] pathway, which is also involved in the pathogenesis of RCC [41]. Recent findings demonstrate that the increased PRR expression can lead to an increase in the activity of these signalling routes in other solid tumours [21], therefore, similar RAS-independent mechanisms should be also taking into account in renal carcinogenetic processes.

PRR can be found in the cell-surface but also in the cytoplasmic organelles. The protein is expressed in the membranes of the endoplasmic reticulum and the Golgi apparatus, from which it can be released as a soluble isoform (sPRR) after cleaving by ADAM19, furin and Site-1 protease [12,42]. The antibody we used for the IHC analysis detects both membrane-bound and soluble isoforms [42,43]. The sPRR is released to the extracellular space and is found in plasma and urine, where it has been described as a potential diagnostic and/or prognostic indicator of cancer presence and progression [21,22]. PRR is also an essential subunit of vacuolar V-ATPase, a protein complex necessary for the acidification of intracellular compartments (such us lysosomes) and for the regulation of autophagy. This is an important homeostatic phenomenon for neoplastic cells to protect them from intracellular stress, and PRR is also involved in tumour cell proliferation and progression by promoting autophagy [12,21,44,45]. Our data show cytoplasmic granular staining of PRR in RCCs, which could be associated with these intracellular phenomena.

PRR has also been described in benign tumours, such as adrenal [46,47] and colorectal adenomas [27]. In agreement with this, our results showed that PRR was expressed in RO and with the same intensity as in ChRCC, tumours that are histogenetically related [6]. These findings suggest that this protein could induce proliferative but non-invasive signals in some tumours. It should also be noted that, in a previous analysis, we observed that neither ACE nor ACE2 were expressed in RO and ChRCC [15], which suggests that the role of PRR in these tumours could be RAS-independent. The degree to which PRR exerts its RAS-dependent or -independent actions in each kidney tumour subtype and the way in which this affects the onset and progression of the tumour is an issue that needs further investigation.

In summary, this study describes PRR protein in a series of kidney tumours with different topographic origins in the nephron. Furthermore, the results in CCRCCs show that the protein expression is increased in both the centre and the invasive front of the most advanced tumours, as has been observed in other solid tumours [21,22,23,24,25,26,27,28,29]. Taken together, this evidence indicates that PRR is associated with renal cancer development and progression. Its potential as a novel biomarker for renal cancer diagnosis and prognosis, but also as a promising therapeutic target, should be taken into account in the future [21].

## Figures and Tables

**Table 1:** Pathological and clinical characteristics of clear cell renal cell (CCRCC) cancer patients. WHO/ISUP: World Health Organization / International Society of Urologic Pathologists. TNM: Tumour (T), Node (N), Metastasis (M). SSIGN: Stage, Size, Grade, and Necrosis. UISS: UCLA Integrated Staging System.

Variables	CCRCC Patients (*n* = 83)
*Diameter*	
≤4 cm	26
>4 to 7cm	37
>7 cm	20
*WHO/ISUP grade*	
Low G1-G2	43
High G3-G4	40
*Local invasion (pT)*	
Organ-confined pT1-pT2	65
Not confined pT3-pT4	18
*Lymph node invasion (N)*	
No	77
Yes	6
*Distant metastasis (M)*	
No	73
Yes	10
*TNM Stage*	
Not-advanced (I-II)	62
Advanced (III-IV)	21
*SSIGN (tumour progression)*	
Low progression	62
High progression	21
*UISS (mortality risk)*	
Low	23
Intermediate	42
High	17
*Patients’ Survival*	
Alive	62
Dead of disease	15
Dead by other causes	6

## Data Availability

Full data will be available from the Corresponding Author upon reasonable request.
